# How Polyphenol Metabolites Spatiotemporally Reprogram Transcription Factors and Human Proteostasis: A Metabolite-Centric Framework

**DOI:** 10.3390/cimb48050529

**Published:** 2026-05-19

**Authors:** José Manuel Pérez de la Lastra, Celia María Curieses Andrés, Elena Bustamante Munguira, Celia Andrés Juan, Eduardo Pérez Lebeña

**Affiliations:** 1 Institute of Natural Products and Agrobiology, CSIC-Spanish Research Council, Avda. Astrofísico Fco. Sánchez, 3, 38206 La Laguna, Spain; 2 Hospital Clínico Universitario de Valladolid, Avenida de Ramón y Cajal, 3, 47003 Valladolid, Spain; cmcuriesesa@saludcastillayleon.es (C.M.C.A.); ebustamante@saludcastillayleon.es (E.B.M.); 3 Deapartment of Organic Chemistry and Cinquima Institute, Faculty of Sciences, Valladolid University, Paseo de Belén, 7, 47011 Valladolid, Spain; celia.andres.juan@uva.es; 4 Sistemas de Biotecnología y Recursos Naturales, 47625 Valladolid, Spain; info@glize.eu

**Keywords:** polyphenols, metabolites, glucuronides, sulphates, urolithins, γ-valerolactones, equol, chronopharmacology, β-glucuronidase

## Abstract

Polyphenols act in humans through authentic metabolites, including regio-isomeric glucuronides/sulphates, O-methylated forms, and microbiota products (urolithins, γ-valerolactones, equol), that reach targets by spatiotemporally gated exposure. Vectorial transport (MRP2/BCRP/P-gp), enterohepatic cycling, and β-glucuronidase hubs create early, surface-proximal microbursts of aglycone/catechol, whereas microbiota metabolites arrive systemically 6–24 h later. Signalling emerges from a continuum of weak noncovalent modulation, conditionally gated electrophile/redox relays (catechol → o-quinone, reversible Michael adduction plus signalling-range H_2_O_2_), and PTM cascades (phosphorylation → acylation → proteostasis) that reprogram NRF2/Keap1, NF-κB/IKK, AMPK/MAPK/PI3K-Akt, SIRT1/HDACs, PPARγ, AhR, and TFEB according to where and when metabolites appear. We provide methods and standards to dose isomer-resolved metabolites at physiological free concentrations (nM-low µM) in transport-competent systems, with PK-informed sampling across seconds–minutes, 15/60/240 min, and 6–24 h, and we outline a research agenda (reference panels, spatial exposure atlases, metabotype-stratified trials, safety windows). Framed this way, polyphenols shift from vague “antioxidants” to programmable dietary signals that enable precision nutrition targeting transcription-factor and proteostasis programmes in vivo.

## 1. Introduction

**Figure 1:**
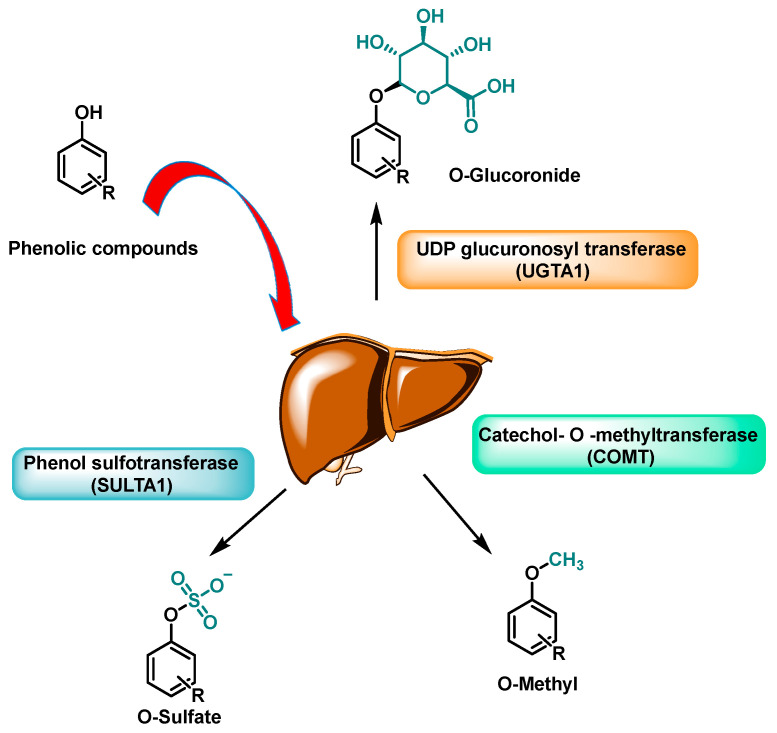
Main hepatic conjugations of bioavailable phenolic compounds mediated by the enzymes UDP glucuronosyl transferase (UGTA1), phenol sulphotransferase (SULTA1), catechol-O-methyltransferase (COMT).

Polyphenols are often portrayed as direct antioxidants, yet at physiologically relevant exposures their most consequential effects stem from bona fide metabolites, host phase II conjugates and microbiota-derived catabolites, that reach targets through routes gated in space and time. After ingestion, UDP-glucuronosyltransferases, sulphotransferases, and catechol-O-methyltransferase convert aglycones into conjugated forms that dominate the circulation, while gut consortia remodel parent scaffolds into smaller xenometabolites, such as urolithins, γ-valerolactones (γ-VLs), and enterolignans, with distinctive transport fingerprints [1], as shown in Figure 1. For a consolidated schematic overview of the complete pathway from polyphenol intake through metabolite routing to transcription-factor engagement and proteostasis reprogramming, see the five-step summary model proposed in Section 8, which is intended as the basis for a future graphical abstract.

Vectorial efflux through MRP2/ABCC2, BCRP/ABCG2, and P-gp/ABCB1, together with enterohepatic cycling and β-glucuronidase hotspots in the gut, inflamed tissues, and tumour microenvironments, is proposed to generate surface-proximal microbursts of aglycone or catechol during the early post-prandial window, followed hours later by the arrival of more permeable microbial products (a model supported by indirect kinetic and transporter-inhibition data but not yet directly imaged in human tissue). These exposure geometries are largely invisible in aglycone-centric monocultures and help account for discordant results across the literature [2].

**Figure 2:**
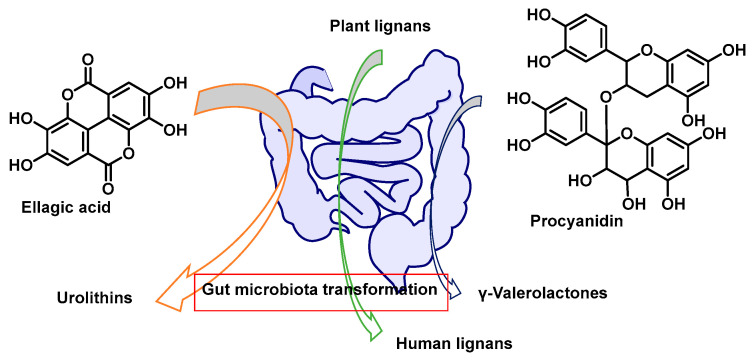
After ingesting foods containing ellagitannins, these are hydrolysed in the stomach to produce ellagic acid. This acid undergoes a series of transformations by the gut microbiota, forming different urolithin molecules. Human microbial degradation of procyanidin B1 dimer provides valerolactone derivatives. Intestinal bacteria transform plant-derived lignans into human lignans.

Mechanistically, signalling spans a continuum: (i) noncovalent modulation of multiprotein assemblies and peripheral enzyme interfaces, (ii) conditionally gated electrophile-redox relays, catechol to o-quinone conversion, reversible Michael adduction, and signalling-range H_2_O_2_ generation, and (iii) downstream post-translational cascades that propagate through phosphorylation, acylation, and ultimately reshape proteostasis [3], Figure 2.

We concentrate on nodes for which convergent preclinical evidence exists, acknowledging that direct target-engagement data in humans remain limited for most at physiologically plausible concentrations, NRF2/Keap1-ARE, NF-κB/IKK, AMPK/MAPK/PI3K-Akt, sirtuins/HDACs (e.g., SIRT1), PPARs (γ), AhR, and TFEB (Transcription Factor EB, master regulator of lysosomal biogenesis and autophagy)-directed lysosomal-autophagy programmes, and delineate which metabolites are operative, when and where they arrive, and the readouts that verify target engagement in intact systems [4]. We advance a metabolite-centric, exposure-aware model in which authentic metabolites, phase II conjugates and microbiota products, and their spatiotemporal delivery govern target access and shape cell- and tissue-level biology. The present article is a narrative mechanistic review with an embedded methodological roadmap. Its specific objectives are threefold: (i) to synthesise the mechanistic evidence linking authentic polyphenol metabolites to transcription-factor and proteostasis reprogramming through noncovalent, electrophile/redox, and PTM-cascade channels; (ii) to map how transporter topology, enterohepatic cycling, and β-glucuronidase hubs create the spatiotemporal exposure geometry that governs which molecular targets are reachable in vivo; and (iii) to translate this framework into tiered experimental standards that can be adopted progressively by laboratories with different levels of resource and technical capability. The review does not perform a systematic meta-analysis; instead it prioritises mechanistic depth and methodological actionability over exhaustive literature cataloguing.

## 2. Chemistry-to-Proteome Mechanisms

This narrative review drew on searches of PubMed, Web of Science, and Scopus conducted between January 2020 and March 2025, with supplementary coverage of key earlier foundational studies. Search terms combined polyphenol metabolite class descriptors (“glucuronide”, “sulphate conjugate”, “urolithin”, “γ-valerolactone”, “equol”) with molecular target terms (“NRF2”, “NF-κB”, “AMPK”, “TFEB”, “SIRT1”, “HDAC”, “AhR”, “PPAR”) and methodological filters (“CETSA”, “chemoproteomics”, “pharmacokinetics”, “transport”). Inclusion was based on four criteria: (1) use of authentic phase II conjugates or microbiota-derived metabolites rather than aglycone surrogates alone; (2) physiologically plausible dosing (ideally with reported unbound concentrations); (3) mechanistic resolution at the level of target engagement, PTM change, or gene expression; and (4) availability in English. Studies relying exclusively on supraphysiological aglycone concentrations were included only when they provided foundational mechanistic context. Given the narrative format, formal PRISMA flow was not applied; instead, the selection logic was guided by mechanistic depth and translational relevance, with priority given to primary research over secondary reviews where possible.

Physiological polyphenol metabolites drive proteome change via three channels: (1) noncovalent biasing of multiprotein assemblies/peripheral enzyme interfaces that redirects ubiquitination, docking, and substrate capture without deep orthosteric binding, (2) conditionally gated covalent–redox events, catechol → o-quinone, reversible Michael adducts, and signalling-range H_2_O_2_, that edit Keap1 and IKK, and (3) PTM cascades (phosphorylation → acylation → proteostasis) that embed cues into durable programmes. Effects are tissue-specific, shaped by transporter topology (MRP2/BCRP/P-gp), enterohepatic cycling, and β-glucuronidase hotspots, and temporally staggered by host-microbial chronopharmacology; thus, we prioritise intact-system engagement (CETSA/TPP, chemoproteomics) and PK-aligned dosing with authentic metabolites at nM–low µM free levels, since ignoring isomer identity, free concentrations, and spatial exposure inflates artefacts and obscures biology [5,6].

### 2.1. Noncovalent Modulation

At physiologically relevant exposure, noncovalent modulation is a plausible and, in several model systems, a well-documented way through which polyphenol metabolites may recalibrate signalling, relying on modest yet strategically positioned interactions that bias multiprotein assemblies and regulate enzyme access rather than occupying deep orthosteric cavities; however, direct biophysical evidence for these interactions in intact human cells or tissues remains sparse [7].

**Figure 3:**
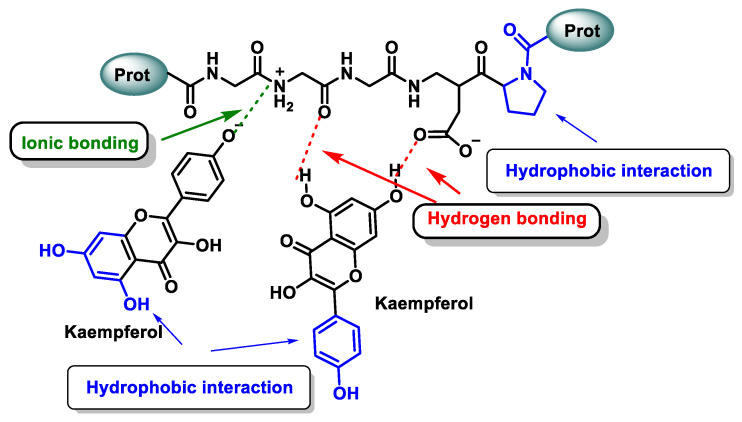
Representation of noncovalent interactions of hydrogen bonds, hydrophobic interactions, and ionic bonds between proteins and polyphenols (Kaempferol).

At nM–low µM exposure, O-methylated/conjugated metabolites retain enough aromatic surface and polarity to fit shallow interfacial pockets; despite sub-mM affinities, they act by stabilising or loosening hinge contacts that gate ubiquitination, kinase docking, and chromatin-enzyme throughput [8], as shown in Figure 3.

In the Keap1-Cul3-NRF2 axis, modest binding at the Kelch pocket or BTB dimer interface can noncovalently lower NRF2 ubiquitination flux, amplified by high local concentrations near apical membranes or deconjugation sites. Similarly, in the IKK complex, small shifts in scaffold stability or subunit positioning reset NF-κB activation thresholds without broadly inhibiting enzymatic catalysis [9].

A second theme is chaperone control: Hsp70/Hsp90 and cochaperones set nuclear availability of TF clients (e.g., AhR, steroid receptors); weak metabolite binding at nucleotide or cochaperone sites tilts client release, altering translocation probability and dwell time. Likewise, HDACs/sirtuins possess peripheral pockets and PPIs that govern substrate capture; hydrophobic–aromatic metabolites there shift apparent Km/Vmax for select acetyl-lysines, biasing transcriptional programmes without engaging catalytic metals or NAD^+^ directly [10].

Noncovalent effects appear as graded, reversible set-point shifts, partial ARE activation, modest NF-κB damping, selective reweighting of sirtuin substrates, that accumulate over hours into transcriptional outputs and intensify where exposure concentrates (engagement methodology: Section 5) [11,12].

### 2.2. Covalent and Redox-Mediated Effects

**Figure 4:**
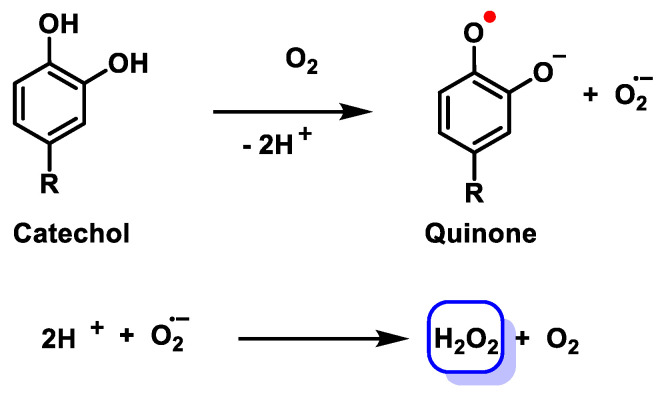
The oxidation of catechol to quinone generates a superoxide radical as a by-product. The superoxide radical undergoes dismutation (2 O_2_^−^ + 2 H^+^ → H_2_O_2_ + O_2_) to yield H_2_O_2_, which is less reactive than superoxide; water is not a reactant in this reaction.

Electrophilic/redox actions arise when catechol/galloyl polyphenols, or locally regenerated aglycones, oxidise to o-quinones that act as soft Michael acceptors and redox relays. These o-quinones form reversible thioether adducts with low-pKa cysteines or glutathione; retro-Michael exchange and thiol-network editing render covalent occupancy transient unless sustained by ongoing quinone formation, as shown in Figure 4.

**Figure 5:**
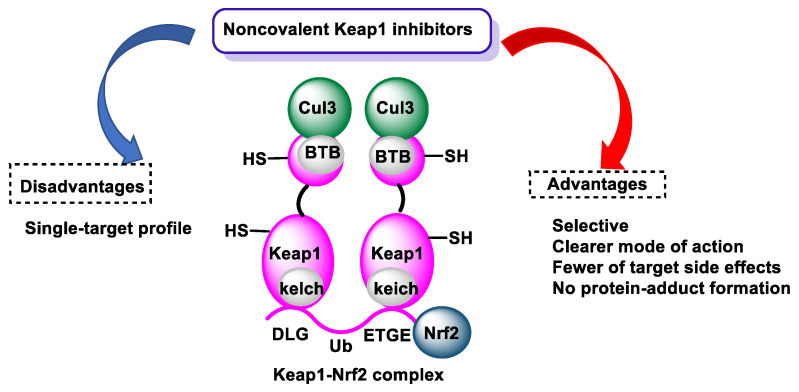
Structure of the Keap1-NRF2 complex. Advantages and disadvantages of noncovalent Keap1 inhibitors.

Quinone–semiquinone cycling generates signalling-range H_2_O_2_, so covalent adduction and oxidant flux often cooperate. Keap1-NRF2 is key: adducting Keap1 cysteines (Cys151, Cys273/288) impairs NRF2 ubiquitination and amplifies ARE programmes. Even when circulating species are conjugated, β-glucuronidase-rich niches (gut, inflamed tissue, tumour microenvironments) regenerate catechols that oxidise locally to quinones, gating NRF2 activation to those compartments [13], as shown in Figure 5.

Second, NF-κB/IKK: redox-sensitive IKKβ cysteines undergo quinone adduction or mild oxidative editing, dampening kinase activity and limiting p65 entry; the effect hinges on glutathione/thioredoxin buffering and MAPK inputs, making biphasic or partial inhibition at physiological exposure expected [14].

Chaperones and deacetylases are redox-sensitive: cysteine-rich Hsp70/Hsp90 cochaperones and Zn-coordinated class I/II HDAC pockets respond to electrophiles, so subtle, reversible occupancy shifts client maturation and chromatin access. SIRT1 is governed mainly via redox–NAD^+^ coupling, with indirect effects under quinone stress. These edits unfold within minutes–hours and are gated by transporter topology (MRP2, BCRP, P-gp limiting electrophile entry) and local deconjugation that creates pericellular “electrophile microfactories” [15].

**Figure 6:**
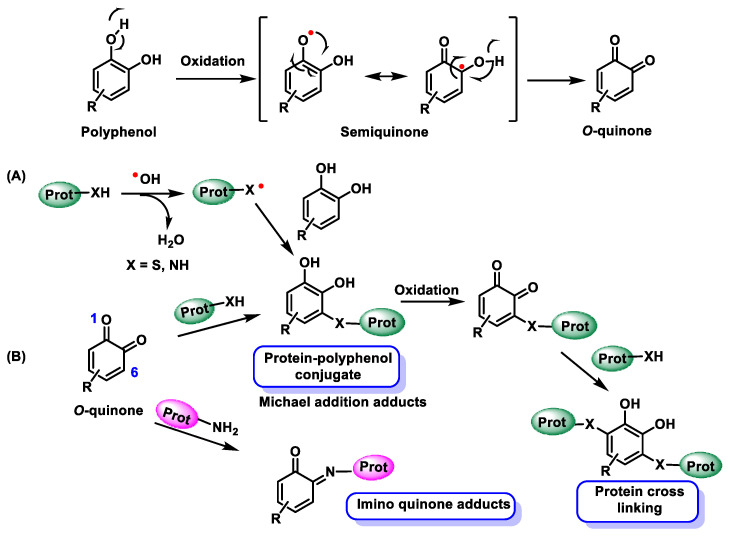
Covalent bonding of polyphenols with proteins. Oxidised polyphenols (o-quinones) react rapidly with amino groups and sulphhydryl groups through a Michael addition reaction, leading to the development of protein–polyphenol adducts. (**A**) Polyphenol–protein complexes formed by free radicals (**B**). Polyphenol–protein complexes formed by oxidation (Michael addition and imino-quinone adducts).

Net result: Catechol → o-quinone conversion provides a gated covalent handle and tuneable oxidant source that recalibrate sensor networks without high systemic aglycones, as shown in Figure 6, (attribution strategy: Section 5.2).

### 2.3. Post-Translational Modification Cascades

PTM cascades translate weak, distributed signals from polyphenol metabolites into durable transcriptional shifts via interconnected phosphorylation, acylation, and proteostasis pathways whose timing and spatial organisation mirror exposure biology [16].

Brief, spatially gated electrophile/H_2_O_2_ pulses from catechol → o-quinone chemistry and β-glucuronidase microbursts trigger 15–60 min AMPK/MAPK/PI3K-Akt phosphorylation waves that rebalance metabolic sensing, stress adaptation, and chromatin access without high-µM occupancy of any single target. Over 1–4 h, lysine acylation is reprogrammed: SIRT1 tracks NAD^+^ and subtle interfacial biasing, while electrophile-tuned class I/II HDAC conformations modulate substrate capture, jointly tilting FOXO/PGC-1α/p65 toward cytoprotective, anti-inflammatory setpoints [17].

In parallel, the ubiquitin–autophagy axis converts the kinase–acetylation dialogue into proteome quality control: TFEB-driven lysosomal biogenesis and selective mitophagy clear damaged organelles and trigger secondary transcriptional programmes, with urolithin A exemplifying late, microbiota-dependent links to mitochondrial quality control and nuclear responses. Timing layers as seconds–minutes for redox/electrophile cues and initial engagement, tens of minutes for kinase propagation, hours for acetylation resets and TF occupancy shifts, and ~4–24 h for transcriptional/proteostatic outputs that outlast the initiating chemical events [18].

With PK-aligned designs and transport-competent models (Section 5), “polyphenol signalling” coheres into a PTM-driven programme whose amplitude and sign are set by which metabolites arrive, where, and when, rather than by supraphysiological aglycone pharmacology [19].

## 3. Who Gets There: Metabolism, Transport, and Microenvironments

In vivo, the biology of polyphenols is governed less by the chemistry of their aglycones than by the metabolites they yield, the routes those metabolites take, and the sites at which they are locally regenerated. This section integrates the exposure determinants that, in turn, shape transcription-factor activity and protein-level effects, ultimately conditioning the character and magnitude of downstream modulation [20].

### 3.1. Phase II Enzymes (UGT/SULT/COMT): Regioselectivity → Metabolite Repertoires with Distinct Target Affinities

After intestinal uptake, polyphenolic aglycones are rapidly processed by UGTs, SULTs, and COMT into position-selective glucuronides, sulphates, and O-methyl ethers that dominate in vivo. This regioselectivity, by dictating which sites are conjugated or O-methyl-capped, rewires charge, H-bonding, pKa, polarity, and transporter recognition, thereby reshaping protein engagement that governs transcription-factor control [21].

Distinct active sites in UGT1A/UGT2B and SULT1/2 steer conjugation to specific phenolic positions (e.g., 3/7-O on flavonoids, 3′/4′ on catechols), while COMT, via Mg^2+^-coordinated, SAM-dependent transfer, adds O-methyl groups that dampen catechol redox cycling and redirect later conjugation routes [22]. The result is a structured repertoire of positional isomers and mixed conjugates (e.g., O-methyl-glucuronides) that differ in complementing shallow protein grooves, biasing multiprotein assemblies, and sustaining redox-coupled signalling [23].

These structural changes have three consequences: (1) the anionic patch from glucuronide/sulphate addition biases binding toward peripheral regulatory pockets over deep orthosteric sites; (2) O-methylation (COMT) dampens catechol redox cycling, but β-glucuronidase-rich niches restore quinone chemistry in situ; and (3) altered transporter recognition concentrates conjugates at apical/canalicular membranes where Keap1 and membrane-proximal kinases are accessible [24,25,26].

Temporal kinetics reflect enzyme interplay: PAPS-limited SULTs dominate at low dose then saturate, higher exposure shifts to glucuronidation and enterohepatic cycling, and O-methylation can occur pre- or post-conjugation, improving hydrophobic fit while dampening redox. Specific sequences (e.g., 3′-O-methylation → 7-O-glucuronidation) produce conjugates with distinct transporter recognition and latent pro-electrophile potential upon local deconjugation [27].

In sum, phase II regioselectivity determines which conjugates form, where they accumulate, and whether aglycones regenerate in situ, thereby bounding protein engagements and the transcriptional programmes realised in vivo [28].

### 3.2. Transporters (MRP2, BCRP, P-gp): Partitioning Across Gut–Liver–Blood, Tissue Gradients, and Intracellular Trafficking

Rather than diffusing freely, phase II conjugates (and residual aglycones) are routed by polarised ABC efflux pumps (MRP2/ABCC2, BCRP/ABCG2, P-gp/ABCB1) across epithelia/barrier endothelium, creating steep apical–basolateral gradients that determine which cells encounter signalling-competent species and when [29].

In the intestine, apical MRP2/BCRP expel anionic glucuronides/sulphates to the lumen while P-gp limits aglycone residence and basolateral escape, producing transient apical enrichment, high luminal loads, and low plasma despite intense epithelial exposure. In hepatocytes, canalicular MRP2/BCRP (±P-gp for lipophilic aglycones) drive biliary excretion, when saturated/impaired, basolateral MRP3 back-exports conjugates to blood, enlarging systemic pools and redirecting exposure to extrahepatic tissues [30].

Kidney proximal tubules and barrier endothelia (BBB, placenta) deploy BCRP and P-gp to lower systemic levels and protect vulnerable sites, biasing transcription-factor effects toward barrier epithelia repeatedly bathed apically by circulating Phase II products, especially conjugates [31].

These polarised systems run on saturable, isoform-specific kinetics tuned by xenobiotic sensors (PXR, CAR, AhR) and stress pathways (NRF2). Inflammation, hypoxia, diet-borne ligands, and common ABCG2/ABCB1 polymorphisms can recalibrate capacity within hours–days, reshaping tissue gradients independent of intake. Basolateral uptake via OATPs (SLCO) and OATs (SLC22) creates uptake → canalicular efflux cycles that amplify biliary concentrations and heighten local reactivation along the biliary tree and intestinal lumen [32].

Overall, a partitioning system concentrates conjugates at apical surfaces and in bile, seeds enterohepatic cycling, keeps circulating levels low unless capacity is exceeded, and intermittently permits basolateral escape, thereby dictating where TF-relevant exposures arise [33].

Vectorial transport thus sculpts gut–liver–blood exposures with high anatomical precision: apical enrichment raises mucosal levels far above plasma; canalicular overload diverts conjugates via MRP3 to extrahepatic tissues; and transporter/SLC fingerprints bias intracellular routing toward cytosolic redox sensors over nuclear targets [31,34].

**Figure 7:**
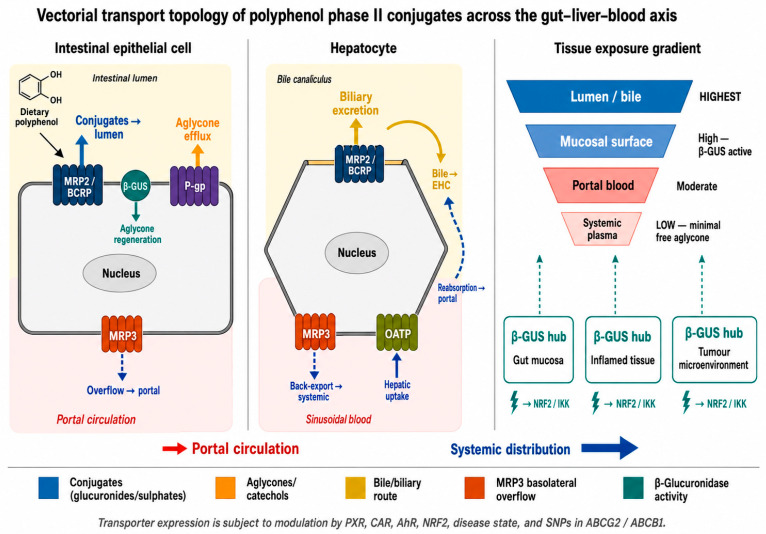
Vectorial transport topology of polyphenol phase II conjugates across the gut–liver–blood axis and its consequences for tissue-specific exposure.

The signalling consequences of polyphenol metabolites are inseparable from the anatomical constraints imposed by polarised ABC efflux transporters, which create steep concentration gradients across the gut, liver, and blood compartments rather than allowing conjugates to distribute freely (Figure 7). Apical and canalicular efflux by MRP2, BCRP, and P-gp concentrates glucuronides and sulphates at barrier surfaces and in bile, while keeping systemic plasma levels modest, and MRP3-mediated basolateral escape provides a regulated overflow route to extrahepatic tissues when canalicular capacity is exceeded. These gradients determine with anatomical precision where β-glucuronidase-dependent aglycone regeneration can occur and therefore where electrophile and redox signalling can be initiated, which is the spatial logic that all subsequent mechanistic discussion in this review depends upon.

### 3.3. Enterohepatic Recirculation and Local Deconjugation: β-Glucuronidase Hubs

Enterohepatic recirculation shapes exposure: hepatic phase II conjugates are secreted into bile, delivered to the gut, and hydrolysed by microbial β-glucuronidases to regenerate aglycones that are reabsorbed or further catabolised. This cycle prolongs residence and creates localised, time-gated aglycone/catechol microbursts at epithelia that activate redox-sensitive proteins and transcriptional programmes without high systemic concentrations [35].

Outside the gut, host β-glucuronidase (GUSB, the lysosomal enzyme encoded by the GUS1 locus) from immune cells or lysosomal leak drives local deconjugation in inflamed tissues and tumours, decoupling plasma from tissue-effective exposure and explaining robust signalling despite low circulating aglycone. Microbial GUS (microbial β-glucuronidase) enzymes, a diverse superfamily with loops favouring aryl-O-glucuronides, operate via a retaining double-displacement and are promoted at mildly acidic–neutral pH typical of the colon and inflamed lesions [36].

Mammalian GUSB, chiefly lysosomal, can be released extracellularly via degranulation or necrosis (including NETs); its activity rises in protease-rich, acidic microenvironments typical of inflammation and cancer [37].

Three β-glucuronidase hubs are proposed to dominate based on current tissue-distribution and activity data, though direct in vivo quantification in humans remains limited: (1) gut lumen–mucosa, where biliary conjugates load mucus and GUS regenerates aglycones that are quickly reconjugated/oxidised to o-quinones, engaging Keap1/IKK, (2) inflamed tissue, where high GUSB and vascular leak create an electrophile-rich, low-pH milieu that primes NRF2 and can damp NF-κB, and (3) tumours, where EPR and abundant GUS let glucuronides act as latent prodrugs, biasing AhR and oxidative-stress programmes in adjacent cells [38].

Enterohepatic recirculation sets a timeline: an early host-conjugate wave, biliary pulses whose β-glucuronidase hydrolysis yields brief mucosal aglycone spikes, and a later 6–24 h rise in microbial catabolites (urolithins, γ-valerolactones); thus, high local GUS can make tissue aglycone exposure exceed plasma readouts. Pericellular deconjugation reconciles low systemic electrophile burden with robust signalling, regenerated catechols adduct Keap1 or temper IKKβ, while redox-derived H_2_O_2_ propagates via AMPK/MAPK signalling cascades [39].

Together, enterohepatic recirculation and β-glucuronidase hubs convert circulating glucuronides into spatially gated electrophile/redox sources that dictate which TF and protein nodes are engaged in vivo (methodological detail: Section 5.6).

### 3.4. Microbiota-Derived Metabolites and Metabotypes: Urolithin/Valerolactone/Equol Producers and Interindividual Variability

Microbiota-derived metabolites create a delayed, systemic wave that explains divergent responses despite similar intakes. After phase II conjugation and apical efflux, colon microbes cleave, dehydroxylate, reduce, and lactonise select scaffolds into smaller xeno-metabolites with altered polarity, redox behaviour, and transporter recognition. Reabsorbed and reconjugated, they still permeate extraintestinal tissues, yielding delayed PK peaks and engaging distinct targets with correspondingly different activity spectra [40].

Three families exemplify this. Urolithins arise when anaerobes convert ellagic acid (from ellagitannins) via lactone opening, decarboxylation, and stepwise dehydroxylation to dibenzopyran-6-one cores (A-D); they circulate mainly as glucuronides/sulphates but can transiently re-form as aglycones at β-glucuronidase-rich sites, activating mitochondrial–lysosomal quality control and dampening inflammatory tone [41].

γ-VL form from flavan-3-ols/procyanidins via C-ring fission, β-oxidation-like side-chain shortening, and lactonisation to 5-(hydroxyphenyl)-γ-VL; subsequent O-methylation and phase II conjugation diversify derivatives whose stereoelectronic features improve noncovalent fit at nuclear receptors and kinase nodes [42].

Equol arises from daidzein via reduction/dehydroxylation, yielding a chiral ER-active ligand with distinct receptor bias. Collectively, these microbial products show delayed T_max_ (≈6–24 h) and longer apparent half-lives than aglycones, creating “late windows” aligning with urolithin A-driven mitophagy/TFEB activation, AMPK/PPAR/AhR tuning by γ-VLs, and ERβ/PPAR-linked programmes mediated by equol/enterolignans [43].

Inter-individual variability reflects stable yet modifiable metabotypes. For ellagitannins, three are recognised, urolithin A-dominant, B/mixed, and non-producers, shaped by microbial gene clusters and ecology (pH, transit time, fibre, antibiotics). For flavan-3-ols, differing C-ring-fission/lactonisation across *Clostridiales*/*Eubacteriales* yields diverse γ-VL regio-isomer ratios that, after host O-methylation/glucuronidation, create distinct transporter fingerprints and differential engagement of receptor and kinase targets [44].

Equol production is limited to individuals harbouring Adlercreutzia/Slackia; non-producers often form O-desmethylangolensin with weaker ERβ selectivity. Urolithin A boosts mitophagy and TFEB-driven lysosomal biogenesis, low-µM γ-valerolactone conjugates recalibrate AMPK/MAPK and PPAR/AhR signalling, and equol/enterolignans preferentially engage ERβ/PPAR with crosstalk to NRF2/SIRT1 nodes. Metabotype-aware work is essential: donor-stratified fermentations/colon simulators, isomer-resolved analytics, PK-aligned metabolomics with TF biomarkers, and transport-competent cells that preserve access constraints. Control confounders include matrix effects, recent antibiotics/PPIs, ABC limits, and equol chirality. Altogether, microbial genotype–phenotype individualises, time-stages signalling and determines which TF/protein circuits are reachable in vivo [45].

### 3.5. Chronopharmacology: Early (Host-Conjugates) vs. Late (Microbial Catabolites) Activation Windows

In vivo, diet-to-signalling is biphasic: early host conjugates and later microbiota catabolites, each with distinct chemistry, PK profiles, spatial reach, transporter coupling, and TF tiers. Post-ingestion, intestinal/hepatic phase II generates glucuronides/sulphates routed apically by MRP2/BCRP and canalicularly in hepatocytes, loading lumen/bile while keeping plasma at modest levels [26].

Vectorial transport creates transient perimembrane pooling at barrier epithelia, β-glucuronidases at the mucus–cell interface (microbial or immune) hydrolyse aryl-glucuronides, generating brief aglycone/catechol bursts that trigger redox sensors and kinase waves without sustained systemic exposure [46].

Enterohepatic cycling creates an early conjugate crest (minutes–hours) followed by biliary pulses whose surface hydrolysis yields brief aglycone spikes, with high β-glucuronidase, tissue aglycone AUC can exceed plasma expectations. This aligns with a cascade: seconds–minutes for electrophile/H_2_O_2_ and first engagements, 15–60 min for AMPK/MAPK waves, 1–4 h for TF-occupancy shifts, and 4–24 h for chromatin/proteostasis outputs that outlast the initiating chemistry [39].

Late-phase exposure is driven by smaller, more permeable colon-derived xenometabolites–urolithins, γ-valerolactones, and equol, that reach circulation with delayed maxima (T_max_ ≈ 6–24 h), show longer apparent half-lives, and, despite circulating largely as conjugates, remain permeable enough to access extraintestinal tissues [40].

The late phase favours organelle/nuclear programmes under sustained low-level exposure: urolithin A drives mitophagy and TFEB lysosomal biogenesis, low-µM γ-valerolactone conjugates recalibrate AMPK/MAPK and PPAR/AhR, and equol/enterolignans bias ERβ/PPAR with crosstalk to NRF2/SIRT1. Because catabolite formation is metabotype-dependent (urolithin A-dominant vs. B/mixed vs non-producers, high vs. low γ-VL converters, equol producers vs. non-producers), the late window’s magnitude, and even its presence, is intrinsically person-specific [47].

Both windows are shaped by transporter topology and local deconjugation, conferring anatomical specificity: apical enrichment and canalicular delivery bathe barrier surfaces, favouring NRF2 priming and context-dependent NF-κB tuning, when canalicular capacity is exceeded or inhibited, MRP3-mediated basolateral escape raises circulating conjugates and exports signalling to extrahepatic tissues. β-Glucuronidase hubs in inflamed and tumour microenvironments regenerate early-like aglycone bursts from host conjugates or late microbial products, aligning TF activation with disease geography rather than bulk pharmacokinetics [48].

Use PK-informed designs: dose authentic metabolites at nM–low µM timed to early/late, sample at seconds–minutes, 15/60/240 min, and 6–24 h, and use transport-competent models with β-glucuronidase co-exposures or colon-simulator effluents. Ignoring this chronopharmacology (single time points or supraphysiologic aglycone boluses) hides true engagement and inflates artefacts; early windows are conjugate-routed, surface-proximal, and electrophile-limited, whereas late windows are microbiota-dependent, more systemic, and programmatic [12].

**Figure 8:**
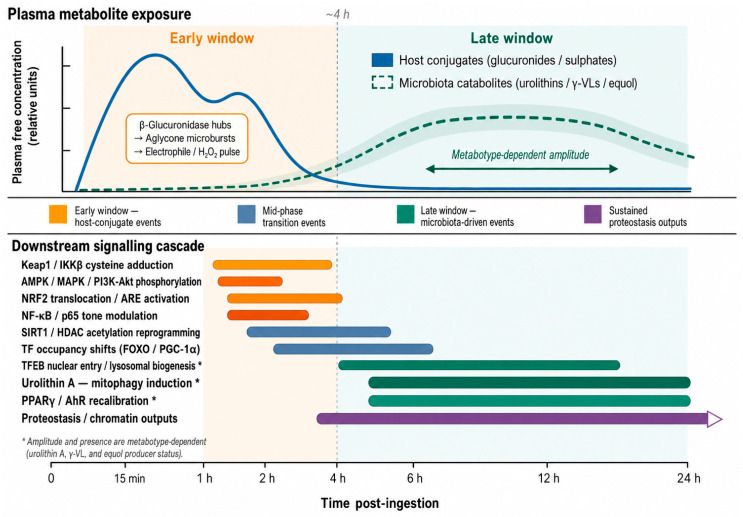
Biphasic chronopharmacology of polyphenol metabolite exposure and its downstream signalling consequences.

The metabolic fate of ingested polyphenols unfolds on two temporally distinct timescales that reflect the different biochemical origins of the operative species (Figure 8). An early window, spanning minutes to approximately four hours post-ingestion, is shaped by host phase II conjugates routed vectorially to apical and biliary surfaces, where β-glucuronidase activity generates transient aglycone microbursts that initiate electrophile and redox signalling. A late window, emerging between six and twenty-four hours, is defined by microbiota-derived catabolites whose appearance, amplitude, and identity depend on individual metabotype. Understanding this biphasic architecture is essential for the correct design and interpretation of any experimental or clinical study of polyphenol bioactivity.

### 3.6. Catechol → o-Quinone Electrophiles: Reversible Michael Acceptors and Redox Relays

The catechol → o-quinone chemistry introduced in Section 3.2 is further shaped by the transport and deconjugation landscape: at β-glucuronidase hubs, heavy conjugate delivery regenerates reactive catechols that oxidise locally to o-quinones, enabling gated electrophile/H_2_O_2_ signalling at barrier and diseased sites without high systemic aglycone. The key steps in this chemistry are: (1) enzymatic or non-enzymatic two-electron oxidation of the catechol to the corresponding o-quinone, a process accelerated by cellular peroxidases or metal ions; (2) semiquinone cycling, in which one-electron reduction in the o-quinone by cellular reductants (notably NQO1’s obligate two-electron reduction provides a competing bypass) produces superoxide as a by-product, which is subsequently dismutated to H_2_O_2_; and (3) reversible Michael adduction of the o-quinone with low-pKa cysteine residues on sensor proteins, creating short-lived thioether adducts that retro-Michael exchange under reducing conditions. The net result is a transient, spatially confined electrophile and oxidant pulse whose amplitude is set by the local balance of catechol delivery (via β-glucuronidase activity), oxidising capacity, and the competing quench supplied by glutathione, thioredoxin, and NQO1. In NQO1-rich parenchyma, adduct lifetimes are curtailed and quinone-mediated H_2_O_2_ generation is attenuated, enforcing hormetic, transient NRF2 activation rather than sustained oxidative stress. Conversely, in tumour microenvironments and chronically inflamed tissue where NQO1 expression may be dysregulated and glutathione pools are depleted, the same chemistry can produce more prolonged sensor engagement. Attribution of observed protein modifications to authentic catechol-derived quinones rather than artefactual air oxidation requires the composite chemoproteomics strategy detailed in Section 5.2, including vehicle controls under strictly anaerobic conditions and isotopically coded nucleophile traps as reference standards [49,50].

## 4. Transcription Factor and Protein Targets: Evidence Map

The Evidence Map ties each node to the operative species, their timing/location, and how engagement is measured (Table 1). Early windows: host conjugates routed by MRP2/BCRP/P-gp; at β-glucuronidase hubs they create electrophile/H_2_O_2_ microbursts that trigger brief phosphorylation waves. Late windows: microbiota catabolites (urolithins, γ-valerolactones, equol) with superior organelle/nuclear access and metabotype-dependent amplitudes [51].

For each target, we prioritise intact-system readouts, CETSA/TPP and chemoproteomics to confirm engagement, DIA/PRM phospho- and acetyl-proteomics with ARE/NF-κB/XRE (xenobiotic-response element)/TFEB reporters to track signalling, and ChIP-seq for occupancy, using nM–low µM free concentrations in transport-competent models that reproduce vectorial efflux and local deconjugation. Framed by exposure, this reconciles disparate findings and standardises PK-informed, metabotype-stratified designs for more decisive, reproducible experiments [52].

### 4.1. NRF2/Keap1-ARE Pathway

State of human evidence: Before detailing each mechanistic node, it is important to contextualise the level of evidence available across the different tiers of biological complexity. In human intervention studies, the most consistently replicated findings concern urolithin A: the TIMELINE trial (Andreux et al., 2019 [53]) demonstrated that oral urolithin A at 500–2000 mg/day is safe and induces a molecular signature of improved mitochondrial and cellular health in skeletal muscle of healthy older adults, including upregulation of mitophagy and autophagy gene transcripts, representing the most direct human evidence for TFEB/mitophagy engagement reported so far. For NRF2-pathway activation, human data remain largely confined to indirect biomarkers (e.g., increased plasma glutathione, reduced F2-isoprostanes) after polyphenol-rich dietary interventions, without direct target-engagement validation. For NF-κB, AMPK/MAPK, SIRT1, PPARs, and AhR, mechanistic evidence in humans is almost entirely absent at the metabolite level; most human studies report inflammatory cytokine or lipid-panel endpoints that are consistent with, but do not prove, the proposed pathway activations. For equol and ERβ, human pharmacokinetic data confirm systemic exposure in producers, but receptor-occupancy or ChIP data in human tissues are not yet available. In aggregate, the mechanistic axes described in the subsequent subsections are well-supported by cell-based and rodent in vivo evidence, but require substantial translational validation before they can be confidently stated as operative in humans at dietary exposures.

The NRF2/Keap1-ARE module is the canonical electrophile/redox translator of polyphenol metabolism into cytoprotective transcription, yet in vivo activation depends on exposure topology and temporal layering. Basally, Keap1 drives Cul3-mediated NRF2 ubiquitination and turnover; electrophile stress or low H_2_O_2_ oxidises/alkylates key Keap1 cysteines, allowing NRF2 accumulation, nuclear translocation, ARE binding, and induction of detoxication/proteostasis genes (e.g., GSH metabolism, NQO1, HO-1), [54].

Within this framework, metabolites, not aglycones, drive NRF2 control: phase II conjugates are vectorially delivered (MRP2/BCRP apically in enterocytes, canalicular pumps in hepatocytes), creating perimembrane niches repeatedly exposed despite modest plasma; at gut mucus, inflamed tissues, or tumours, local β-glucuronidases regenerate catechols that oxidise to o-quinones, producing localised electrophile/H_2_O_2_ microbursts that engage Keap1 on site [55].

Keap1 has multiple reactive cysteines whose modification hierarchies confer stimulus selectivity: Cys151 (BTB) and Cys273/288 (IVR) are electrophile-sensitive, while other residues preferentially sense H_2_O_2_ via distinct chemistries; together, these inputs tune the rate of NRF2 ubiquitination rather than abolishing it [56].

Catechol-derived quinones act as soft Michael acceptors on cysteines and, via semiquinone cycling, generate signalling-range H_2_O_2_. Together they tilt Keap1-NRF2 without high systemic aglycone. NQO1 induction back-reduces quinones to hydroquinones/catechols, shortening adduct lifetimes and enforcing hormetic, transient activation rather than persistent damage [57].

Anatomy governs NRF2: apically bathed barrier epithelia are primed as transporters and β-glucuronidase concentrate precursors and enable local electrophile generation, whereas NQO1-rich parenchyma mounts briefer, buffered responses. If canalicular capacity is exceeded/inhibited, MRP3 raises circulating conjugates and exports signalling to extrahepatic tissues, eroding spatial restriction. Hence robust ARE activation is observed in models preserving vectorial transport/deconjugation versus weak effects in simple monocultures dosed with non-physiological aglycone boluses [58].

NRF2/Keap1-ARE activation is thus not generic antioxidant activity but a spatiotemporally gated response shaped by transporter delivery, local deconjugation, and tuneable quinone/H_2_O_2_ signalling (engagement methodology: Section 5) [6].

### 4.2. NF-κB/IKK Axis

Polyphenols modulate NF-κB/IKK via weak interface biasing plus gated electrophile/redox inputs, with transporter routing and microenvironments deciding inhibition versus subtle tone-tuning. In resting cells, IKK (IKKα/IKKβ/NEMO) phosphorylates IκB to enable p65/p50 nuclear entry, so small shifts in assembly or catalytic competence can disproportionately affect downstream transcription [59].

Polyphenol metabolites (glucuronides, sulphates, O-methylated) rarely bind deep orthosteric sites, yet, even with sub-mM affinity, they subtly tighten or loosen scaffolds/docking interfaces, shifting activation thresholds without global suppression. These noncovalent set-point shifts are most evident in epithelia apically bathed in conjugates, where transporter topology concentrates exposure at the plasma membrane and perinuclear zones rich in IKK and its upstream adaptors [60].

Beyond noncovalent tuning, catechols regenerated at β-glucuronidase hubs oxidise to o-quinones that Michael-adduct IKKβ cysteines, while semiquinone cycling yields signalling-range H_2_O_2_; together they damp kinase flux and p65 entry in a reversible, buffer- and MAPK-dependent manner. Because conjugates dominate circulation, electrophile chemistry is spatially gated to gut mucus, inflamed lesions, and tumours, where local deconjugation/oxidation forms “electrophile microfactories” that tune NF-κB despite low plasma aglycone levels [61].

Transporters set the tissue map: apical/canalicular efflux (MRP2/BCRP, P-gp) loads lumen/bile while keeping plasma low, if canalicular routes saturate or are blocked, basolateral MRP3 raises circulating conjugates, exporting signalling to extrahepatic sites. This routing explains stronger NF-κB dampening in barrier/inflamed mucosa but weaker effects in protected compartments with strong efflux and limited deconjugation capacity (the full transporter topology and β-glucuronidase hub logic are developed in Section 2.2 and Section 2.3) [62].

Thus, the blanket claim that “polyphenols inhibit NF-κB” is broadly true but mechanistically diverse, spanning noncovalent interface biasing, quinone adduction of redox-sensitive cysteines, and H_2_O_2_-driven crosstalk, whose dominance is set by metabolite localisation and the timing of catechol regeneration across early host-conjugate and later microbiota phases, i.e., the exposure choreography across windows [63].

NF-κB/IKK is thus tuneable, with magnitude and direction set by transporter-shaped delivery, local deconjugation, and redox buffering (engagement methodology: Section 5) [64].

### 4.3. AMPK/MAPK/PI3K-Akt

The AMPK/MAPK/PI3K-Akt network is the cell’s first phosphorylation tier, translating modest polyphenol cues into system-wide shifts in metabolism, stress adaptation, and growth. Engagement reflects rapid, spatially gated redox–electrophile inputs plus slower, metabotype-dependent exposure. Early on, β-glucuronidase-driven aglycone/catechol microbursts oxidise to o-quinones and generate signalling-range H_2_O_2_; the resulting 15–60 min phosphorylation waves (AMPK, MAPKs, PI3K-Akt) are consistent with, but not exclusively attributable to, H_2_O_2_-mediated mechanisms, as additional routes including quinone-mediated phosphatase inhibition and direct electrophile adduction of kinase regulatory subunits may contribute in parallel and cannot be excluded on current evidence [65].

Transporter architecture (MRP2/BCRP/P-gp) anatomically confines these transients: conjugates pool at apical/canalicular membranes, creating perimembrane deconjugation/oxidation hotspots; kinase engagement peaks in barrier epithelia and is muted in protected sites (see Section 2.2) [66].

Outputs are graded, not binary: partial AMPK activation, modest ERK/JNK rebalancing, and context-dependent Akt modulation; amplitude depends on local redox buffering (GSH/thioredoxin), concurrent inputs (e.g., IKK/NF-κB), and uptake routes enabling intracellular access by conjugates or transiently regenerated aglycones [67].

A delayed layer emerges as microbial catabolites enter circulation: γ-VLs from flavan-3-ols (mainly low-µM phase-II conjugates) finely tune AMPK/MAPK with PPAR/AhR crosstalk, while urolithin A from ellagitannins drives mitophagy and TFEB-centred lysosomal programmes that secondarily rewire AMPK/MAPK signalling nodes [68].

Because catabolite emergence and amplitude vary by metabotype (high vs. low γ-VL converters, urolithin A producers vs. non-producers), the late phosphorylation landscape is person-specific: identical intakes can yield divergent AMPK/MAPK/Akt trajectories driven by microbial competence and xeno-metabolite timing (T_max_ ≈ 6–24 h). These delayed inputs favour organelle-centric adaptations, mitochondrial quality control, lysosomal biogenesis, and selective autophagy, that reset kinase set points and prolong transcriptional effects beyond the initiating chemical events [39].

Under PK-aligned, transport-competent conditions (Section 5), the AMPK/MAPK/PI3K-Akt tier behaves as a temporally stratified, spatially gated integrator channelling polyphenol chemistry into coherent metabolic and stress responses [69].

### 4.4. Sirtuins/HDACs

Sirtuins and HDACs, especially SIRT1, form the acetylation layer translating faint polyphenol signals into durable transcriptional programmes. Effects depend less on tight orthosteric binding than on redox/NAD^+^ coupling, subtle peripheral-interface modulation, and anatomy-driven timing/location of metabolite encounters in vivo, i.e., exposure [70].

At physiological levels, conjugated/O-methylated metabolites rarely bind SIRT1’s catalytic core with nanomolar potency, instead they act indirectly, tuning NAD^+^ and weakly engaging peripheral interfaces that modulate substrate capture, thus reweighting FOXO, PGC-1α, and p65 deacetylation toward stress-resilient, mitochondriogenic, anti-inflammatory setpoints [71].

Class I/II HDACs have electrophile-sensitive, Zn-coordinated motifs whose conformational balance shifts with local redox/electrophile flux, yielding subtle, widespread chromatin-accessibility changes that amplify sirtuin programmes without metal chelation. These acetylation edits trail AMPK/MAPK/PI3K-Akt waves by ~1–4 h, forming the PTM relay’s mid-phase that integrates transient kinase cues into durable chromatin outputs [72].

Transporter topology gates the magnitude and spatial footprint of SIRT1/HDAC effects via the same MRP2/BCRP/P-gp partitioning and β-glucuronidase hub activity described in Section 2.2 and Section 2.3, creating brief pericellular electrophile/H_2_O_2_ pulses that modulate HDAC conformers and, indirectly, sirtuin signalling networks [17].

Where NQO1 capacity is high, quinone lifetimes are curtailed and signalling collapses into hormetic, transient adjustments rather than damage, helping to explain why barrier epithelia often display SIRT1-compatible deacetylation patterns despite low global aglycone exposure. Methodologically, this argues for transport-competent models with β-glucuronidase co-exposures to connect extracellular dosing to intracellular acetylation shifts, alongside targeted acetyl-proteomics (PRM panels) and locus-specific ChIP-seq to assign function to defined FOXO, PGC-1α, and p65 sites [73].

A delayed, metabotype-dependent layer further modulates sirtuin and HDAC tone. Hours after ingestion, urolithin A promotes mitochondrial quality control via mitophagy and TFEB-centred lysosomal programmes, secondarily increasing NAD^+^ turnover and aligning with SIRT1-leaning transcription. γ-VLs recalibrate AMPK/MAPK and PPAR/AhR axes, supplying upstream cues that converge on sirtuin substrates, and equol/enterolignans bias ERβ/PPAR-driven transcription with crosstalk into NRF2/SIRT1 nodes [18].

Metabotype-driven differences in production and timing mean equal intakes can yield distinct acetylation trajectories and transcriptional outputs, which are variability masked when studies use supraphysiologic aglycone boluses instead of authentic metabolites [74].

When PK-aligned designs are applied (Section 5), SIRT1 and HDACs resolve as integration hubs converting transporter-shaped delivery and organellar feedback into coherent, tissue-specific deacetylation programmes [75].

### 4.5. Aryl Hydrocarbon Receptor (AhR)

AhR bridges metabolic sensing and immune regulation; in polyphenol contexts, activation follows a metabolite-centric, spatially gated model-driven not by nanomolar aglycone binding but by authentic phase-II conjugates and, especially, microbiota-derived catabolites that reach extraintestinal tissues on delayed timelines set by distinctive pharmacokinetics [76].

γ-VLs from flavan-3-ols align with AhR-linked outputs alongside AMPK/PPAR tuning, consistent with modest-affinity ligands gaining leverage via sustained low-µM late-window exposure and favourable transporter/uptake fingerprints. Urolithin A primarily drives mitophagy/TFEB but can crosstalk into AhR-shaped inflammation, while equol is mainly ER-biased yet intersects the immune–metabolic circuitry converging on AhR targets [4].

Because the emergence and amplitude of these xenometabolites are metabotype dependent, two individuals consuming the same dose can display markedly different AhR trajectories solely due to microbial competence and timing (T_max_ ≈ 6–24 h)–a variability that collapses in reductionist assays that over-rely on aglycones [77].

Vectorial efflux via MRP2/BCRP/P-gp concentrates host conjugates at apical membranes and into bile (Section 2.2), and local β-glucuronidase activity (Section 2.3) regenerates aglycones/catechols that generate o-quinones and H_2_O_2_, tilting transcription toward oxidative-stress and AhR signalling in barrier and diseased tissues [33].

In tumours, leaky vasculature and abundant β-glucuronidase reactivate aryl-glucuronides in situ, engaging AhR immunomodulatory programmes despite low circulating aglycone, decoupling tissue signalling from plasma readouts and amplifying local effects at constant exposure levels [37].

When canalicular capacity is saturated or pharmacologically inhibited, basolateral escape, principally via MRP3, elevates circulating conjugate pools and exports signalling competence to extrahepatic tissues, partially dissolving spatial restriction and helping to explain heterogeneous outcomes across models with differing transporter integrity [78].

Mechanistically, AhR sits in a cytosolic chaperone complex whose equilibria are tuned by noncovalent interface biasing and ambient redox tone. Hydrophobic–aromatic metabolites modestly stabilise receptor–cochaperone assemblies or shift nuclear import–export, while H_2_O_2_ pulses from catechol chemistry shape c-Jun/NF-κB crosstalk, conditioning AhR transcriptional output [79].

AhR is thus a context-dependent integrator whose output is tuned by transporter delivery, local deconjugation/oxidation, and individual metabotype, most evident at barrier and tumour-adjacent sites during late, microbiome-dominated windows (engagement methodology: Section 5) [80].

### 4.6. PPARs

Peroxisome proliferator-activated receptors (PPARs), with PPARγ foregrounded, mediate the intersection of lipid metabolism, inflammation, and cellular differentiation, and their engagement by dietary polyphenols follows a metabolite-centric, spatially gated paradigm shaped by conjugation, transport, and local deconjugation rather than by sustained, high-affinity occupation of the receptors by plant aglycones [81].

At physiological levels, authentic conjugates and O-methylated metabolites show only modest affinity for the canonical PPAR pocket, yet their hydrophobic–aromatic surfaces fit shallow grooves and peripheral clefts. Functional leverage comes from weak, distributed contacts that tune coactivator–corepressor exchange and chromatin access, alongside kinase/redox cues that shift PPAR phosphorylation and cofactor availability [82].

Decisive late-window inputs come from microbiota-derived xenometabolites: γ-valerolactones from flavan-3-ols modulate AMPK/MAPK with parallel PPAR and AhR engagement at low-µM, largely conjugated levels, consistent with sustained, systemically accessible exposure. O-methylation and phase-II conjugation diversify receptor fit and transporter recognition, governing tissue reach [42].

Urolithin A, primarily tied to mitophagy/TFEB, secondarily shifts metabolism toward PPAR-aligned transcription, while equol and enterolignans deliver ER-biased signals that spill into PPAR outputs via shared coregulators and metabolic crosstalk [83].

Anatomical routing sharpens selectivity via the same MRP2/BCRP/P-gp and β-glucuronidase logic detailed in Section 2.2 and Section 2.3: conjugates accumulate at apical/canalicular surfaces, locally generated o-quinone/H_2_O_2_ retune upstream kinases that set PPAR phosphorylation and cofactor dynamics, and MRP3-driven basolateral escape extends PPAR engagement to extrahepatic tissues when canalicular capacity is saturated [26].

These spatial constraints help to explain why PPARγ-aligned readouts are most consistently observed in intestinal, hepatic, and inflamed epithelial settings, yet appear inconsistent in reductionist monocultures that lack transporter polarity or local deconjugation [84].

Inter-individual variability tracks metabotypes: γ-VL “high converters” and equol producers show distinct amplitude/selectivity of PPAR programmes vs. non-producers, while a urolithin A-dominant profile drives organelle-centric adaptations that feedback on PPAR nodes. Thus, the late-phase phosphorylation/transcriptional landscape, AMPK-primed coactivator recruitment and PPARγ-dependent adipogenic/anti-inflammatory sets, is person-specific, often eclipsing direct effects of early host conjugates [85].

Under metabotype-stratified, transport-competent conditions (Section 5), PPARγ acts as an integration hub translating sustained microbiota-shaped exposure into coherent lipid–inflammatory programmes [45].

### 4.7. TFEB and Autophagy/Mitophagy

The TFEB-centred lysosomal programme, integrated with bulk autophagy and selective mitophagy, constitutes the proteostatic layer through which polyphenol metabolites translate faint, spatially gated chemical cues into lasting organelle remodelling and transcriptional reprogramming, and its engagement adheres to the biphasic exposure logic outlined above [86].

Early on, transporter-driven delivery of host conjugates to apical/canalicular surfaces creates perimembrane niches; at β-glucuronidase hotspots (mucus, inflamed tissue, tumours), aryl-glucuronides are deconjugated, regenerating aglycones that undergo catechol → o-quinone cycling with concurrent H_2_O_2_ signalling. These inputs bias AMPK/MAPK and lysosomal mTORC1 sensing, promote TFEB dephosphorylation/nuclear entry, and increase autophagic flux [87].

Practically, these microbursts do not depend on elevated plasma aglycone; they arise from vectorial routing of conjugates by MRP2/BCRP/P-gp and localised hydrolysis–oxidation at β-glucuronidase hubs (Section 2.2 and Section 2.3), which is why barrier epithelia and diseased tissues are privileged sites for TFEB engagement under physiological dosing [88].

TFEB functions as a lysosomal nutrient/stress sensor, integrating AMPK/MAPK and redox cues to trigger autophagy, coordinating lysosomal biogenesis, p62 turnover, and selective cargo clearance, then secondarily remodelling nuclear programmes that outlast the initiating chemistry. This PTM relay (seconds-minutes for electrophile/H_2_O_2_, 15–60 min for kinase waves, 1–4 h for acetylation/TF occupancy) places TFEB-driven effects in the 4–24 h window where proteostasis signatures accumulate [89].

The late phase is shaped predominantly by microbiota-derived xeno-metabolites, with urolithin A as the exemplar: appearing systemically around 6–24 h after ingestion and circulating chiefly as conjugates, it is repeatedly linked to mitophagy induction and TFEB-driven lysosomal biogenesis, thereby strengthening mitochondrial quality control and feeding back onto nuclear transcription [90].

Conjugated urolithins act as transportable reservoirs that, at β-glucuronidase-rich sites, are hydrolysed to aglycones with superior organelle access, linking enterohepatic cycling and local deconjugation to organelle-centric adaptation. The magnitude, even the presence, of this late TFEB/mitophagy wave is metabotype-dependent (urolithin A-dominant vs. B/mixed vs. non-producers), explaining inter-individual variability and necessitating metabotype-stratified models and cohorts accordingly [91].

Framed metabolite-centrically, TFEB-driven autophagy/mitophagy are programmatic endpoints of transporter-shaped delivery, local deconjugation, and microbiome-conditioned exposure (endpoints and chronopharmacological sampling: Section 5) [92].

### 4.8. Estrogen-Related Receptors and FOXO Nodes

Polyphenol metabolites engage estrogen-related and FOXO pathways through two convergent mechanisms: microbiota-derived ligands that bias signalling at estrogen receptors, most notably Erβ, and downstream control of FOXO acetylation states mediated by sirtuins and HDACs, a coordination that ultimately hinges on SIRT1/HDAC tuning [93].

In vivo, late-window signals from equol and enterolignans, microbiota-derived and circulating mainly as permeable conjugates, drive ER activity with receptor biases distinct from isoflavone precursors, coupling it to metabolic and inflammatory tone with spillover into PPAR and SIRT1/FOXO circuits that shape downstream pathways [94].

Equol production is limited to some adults; others form O-desmethylangolensin with weaker ERβ bias-making ER-linked outcomes intrinsically metabotype-dependent and explaining inter-individual transcriptional differences at matched intake. Urolithin A chiefly drives TFEB mitophagy and secondarily reshapes FOXO-linked nuclear programmes, while low-µM conjugated γ-valerolactones recalibrate AMPK/MAPK and PPAR/AhR, yielding upstream cues that converge on FOXO acetylation and ER coactivator usage [47].

Spatial gating heightens selectivity: MRP2/BCRP/P-gp route conjugates apically/canalicularly, repeatedly bathing mucosa, β-glucuronidase creates pericellular aglycone microbursts that tune ER/FOXO despite low circulating aglycone. If canalicular capacity is exceeded, MRP3-mediated basolateral escape exports signalling to extrahepatic sites [95].

PK-aligned dosing of authentic metabolites at nM–low-µM free levels, enantioselective equol assays, ER/ERE reporters with ChIP occupancy, and FOXO metrics tied to SIRT1 in transport-competent models with β-glucuronidase co-exposures can be used to recreate spatial gating [96].

Viewed this way, estrogen-related and FOXO nodes form a late, metabotype-conditioned, anatomically gated module integrating ER-biased ligands with SIRT1-driven FOXO deacetylation to steer anti-inflammatory and metabolic transcriptional programmes [97].

The mechanistic basis for ER/FOXO engagement rests on the same exposure logic developed in Section 2, Section 3.1, Section 3.2, Section 3.3, Section 3.4, Section 3.5 and Section 3.6. Equol and enterolignans reach circulation with delayed Tmax (6–24 h) predominantly as sulphate and glucuronide conjugates, and their modest affinity for ERβ’s ligand-binding domain is amplified by sustained low-level tissue exposure rather than transient high peaks. At β-glucuronidase-rich surfaces, mucosal epithelia and inflamed lesions, local hydrolysis releases the aglycone transiently, enabling direct ERβ interaction and downstream coactivator recruitment without imposing a systemic hormonal burden. FOXO factors (FOXO1, FOXO3a) are brought into this circuit through SIRT1-mediated deacetylation: AMPK and PI3K-Akt phosphorylation events that follow early electrophile/H_2_O_2_ cues set the redox and NAD+ environment in which SIRT1 activity is permissive, tilting FOXO occupancy at stress-response and metabolic gene loci toward cytoprotective setpoints [98].

Inter-individual variability in this module is therefore compounded: equol producer status (driven by Adlercreutzia/Slackia colonisation), the amplitude of AMPK/SIRT1 signalling in the early window, and the degree of local β-glucuronidase activity at target tissues all modulate the net ER/FOXO transcriptional output. Non-producers who nonetheless exhibit enterolignans may still engage FOXO through the SIRT1 arm if the early kinase–redox cascade is intact, illustrating how the two convergent mechanisms can operate with some independence. Methodologically, this argues for enantioselective analytics for equol and its precursors, FOXO nuclear localisation assays combined with acetylation-site proteomics (particularly FOXO1 Lys242/245/262 and FOXO3a Lys242/245), and matched ERβ chromatin immunoprecipitation to distinguish direct receptor occupancy from indirect SIRT1-mediated chromatin remodelling. Transport-competent models with β-glucuronidase co-exposure, stratified by equol producer status, are essential to avoid collapsing the inter-individual variability that defines this node in vivo [99].

## 5. Methods and Standards

This chapter translates the metabolite-centric, exposure-aware framework into practical guidance for designing/reporting studies that trace polyphenol signals to authentic metabolites, positional glucuronides/sulphates, O-methylated derivatives, and microbiota products (urolithins, γ-valerolactones), tested at physiologically relevant free concentrations (nM-low µM) [100]. To avoid redundancy with Section 3 and Section 4, recurring concepts (vectorial transport, β-glucuronidase co-exposure, PK-aligned windows) are cited by section reference rather than re-explained in full; readers are directed to those sections for mechanistic context.

To make this framework practically useful, methods are organised into three tiers. The minimal tier comprises the essential steps accessible to most laboratories: dosing authentic metabolites at reported unbound concentrations in standard polarised epithelial models (e.g., Caco-2/TC7 with verified ABCC2/ABCG2 expression), using commercially available CETSA as primary target-engagement readout, and sampling phosphorylation and gene-reporter endpoints at two time points aligned to early (60 min) and late (6–24 h) windows. The recommended tier adds isomer-resolved materials verified by LC-HRMS, competitive cysteine-directed ABPP, targeted phospho/acetyl PRM panels, and β-glucuronidase co-exposures in transport-competent models. The frontier tier includes isoTOP-ABPP with isotopically coded nucleophile traps, gut–liver microphysiological systems, multiplexed MS-TPP across entire protein networks, and PBPK-guided dosing with metabotype stratification. We prioritise intact-system engagement (CETSA/TPP, chemoproteomics) before mechanism, and synchronise readouts with chronopharmacology (early host conjugates vs. late microbial catabolites) and spatial gating (MRP2/BCRP/P-gp, β-glucuronidase hubs). We require isomer-/enantioselective materials, rigorous free-fraction and intracellular-free quantification, and matrix controls (binding, plastics, redox) to prevent artefacts from supraphysiological aglycone boluses or non-polarised cultures [101].

Operationally, the chapter lays out a bench-to-claims pipeline: (i) dose authentic metabolites in transport-competent, polarised systems and gut–liver micro-physiological models, (ii) demonstrate site-level engagement with competitive ABPP and isotopically coded nucleophile traps, linking occupancy to protein stability by TPP, (iii) chart PTM cascades with time-stamped phospho- and acetyl-proteomics, (iv) connect these signals to genomic outputs (reporters, RNA-seq, ATAC/ChIP) sampled over seconds-minutes, 15/60/240 min, and 6–24 h, and (v) embed all designs within a PK/PBPK framework, stratified by metabotype and transporter status. Reporting standards, exposure provenance, isomer identity, free versus nominal dose, redox/NQO1 controls, and pre-registered endpoints, are specified to ensure comparability, reproducibility, and translational relevance, enabling rigorous evaluation of TF/proteostasis reprogramming in vivo [102].

### 5.1. Use Authentic Metabolites (Glucuronides, Sulphates, Urolithins, γ-VL) at Physiological Concentrations (nM-Low µM)

Give priority to authentic metabolites, positional glucuronides and sulphates, O-methylated derivatives, and bona fide microbiota products such as urolithins and γ-VL, rather than the parent aglycones [103].

Dose at physiologically relevant unbound levels; available human pharmacokinetic data indicate that plasma free concentrations of most glucuronide and sulphate conjugates fall predominantly in the sub-μM to low-nM range, with only a subset of metabolites (e.g., quercetin-3-glucuronide, epicatechin sulphates) reaching low-μM free concentrations after high dietary intake (see Williamson & Clifford 2025 [28] and Carregosa et al. 2022 [51] for representative values; a comprehensive reference table of reported unbound human concentrations per metabolite class is warranted and should accompany any updated version of this framework). Report nominal dose and estimated free fraction, explicitly accounting for serum-protein binding, plastic adsorption, and lipid partitioning. Use isomer-/enantioselective materials with clear structural assignments (3′ vs. 4′ γ-VL, 3-O vs. 7-O glucuronides, equol enantiomers) verified by LC-HRMS and NMR. Confirm stability over the assay window (oxidation/hydrolysis) and monitor inadvertent β-glucuronidase/esterase activity that could regenerate aglycones [104].

In cell models, confirm transport competence (MRP2, BCRP, P-gp) and report medium factors shaping exposure (protein binding, pH, redox-GSH/GSSG), use matrix-matched spike-recovery to verify delivered dose. Align dosing with chronopharmacology, early host conjugates vs. late microbiota metabolites, and sample accordingly. For mechanism, add β-glucuronidase co-exposures to reveal in situ electrophile signalling. Avoid supraphysiologic aglycone boluses, if used as comparators, justify and bracket them with authentic metabolite conditions [105].

### 5.2. Chemo-Proteomics

Map target engagement at the site level using competitive, cysteine-reactive activity-based protein profiling (ABPP) tailored to reversible Michael addition chemistry. In intact, transport-competent cells, pre-incubate with authentic metabolites (nM to low µM), with or without β-glucuronidase co-exposure, then deliver a brief pulse of a broad cysteine-directed probe (e.g., IA-alkyne) [6].

Loss of probe labelling provides a direct readout of occupied or otherwise reactive sites, which should be quantified by LC-MS/MS using isotopic or TMT multiplexing approaches (employing isoTOP-ABPP when available). Incorporate pulse–chase designs to resolve retro-Michael exchange and adduct lifetimes, and run time courses (5-60-240 min) to coincide with early kinase signalling waves. For transient quinones, use isotopically coded nucleophile traps (e.g., NAC-alkyne/DTB) to capture adducts directly, then enrich by CuAAC click chemistry under rigorous controls (vehicle, aglycone comparators, probe-only) [106].

Integrate CETSA or TPP to connect site occupancy with protein stabilisation or destabilisation, prioritising nodes within the Keap1, IKK, chaperone, and HDAC networks. Disentangle H_2_O_2_-mediated responses from true covalent engagement by co-treating with catalase or thiourea, or by modulating NQO1. Rigorously manage artefacts by preventing air- and metal-catalysed oxidation, validating probe chemoselectivity, and reporting both the free fraction and the medium’s redox capacity [107].

UseSP3 (single-pot solid-phase-enhanced sample preparation) or similar paramagnetic clean-up, control FDR at the PSM level, and apply peptide-centric statistics. Annotate modified residues with structural context (solvent exposure, catalytic environment, cysteine pKa). Stratify by metabotype when relevant, and validate priority sites using recombinant proteins via BLI/ITC ± site-directed mutants to close the chemistry-to-function loop [108].

For target engagement, use CETSA/TPP as the primary readout in intact transport-competent cells (±β-glucuronidase, time courses 5/60/240 min, concentration-response), with multiplexed MS-TPP across Keap1, IKK, chaperones, HDACs and SIRT1 [109]. Pair with limited-proteolysis MS for weak-ligand interface shifts; disentangle direct engagement from indirect H_2_O_2_ effects via catalase/thiourea or NQO1 modulation; link occupancy to function through matched reporters (ARE, NF-κB, XRE, TFEB), sentinel PTMs (AMPK ACC-P), and phenotypes (p62 flux, FOXO localisation). Claims should survive isomer swaps and persist under CRISPR/degron loss-of-function tests [110].

### 5.3. Phospho/Acetyl-Proteomics

For phosphorylation, enrich peptides by IMAC or TiO_2_ and quantify using DIA or TMT-coupled TPP/DDA workflows, sampling at 5, 15, 60, and 240 min to capture early kinase waves (AMPK, MAPKs, PI3K-Akt). For acetylation, immunoenrich ε-acetyl-Lys and sample at 1–4 h to cover the mid phase and 6–24 h to span the late microbiota window. Dose authentic metabolites at physiologically free concentrations (nM to low µM) in transport-competent models, with or without β-glucuronidase co-exposure, and confirm intracellular unbound levels by LC-MS in matched wells [111].

Link discovery to function with targeted PRM panels tracking sentinel phospho/acetyl sites (AMPK Thr172, ACC Ser79, ERK/JNK, Akt Ser473/Thr308, FOXO, p65, PGC-1α, H3K9ac/H3K27ac). Infer pathway activity viaKSEA (kinase-substrate enrichment analysis)/motif analysis and integrate with CETSA plus chemoproteomics to tie site changes to bona fide engagement. Distinguish H_2_O_2_ responses from covalent effects using catalase/thiourea or NQO1 modulation, and include β-glucuronidase co-exposures to unmask latent electrophile signalling [112].

Quantification should use spike-ins, matched protein → peptide loads, PSM-level FDR, TMT balance checks, and batch correction; report isomer identity, nominal vs. free dose, medium redox (GSH/GSSG), and plastic losses. Add subcellular fractionation/organellar proteomics when access matters. Validate priority PTMs with orthogonal assays and deposit raw data with annotated sites. Interpret PTM trajectories via early–late chronopharmacology, avoiding single endpoints or supraphysiological aglycone boluses [113].

### 5.4. Genomic Readouts

Align genomic measurements with exposure biology and spatial gating from the outset. Begin with mechanistic reporters, ARE, NF-κB, XRE, TFEB, PPAR/PPRE, and ERE/FOXO, in transport-competent models, with or without β-glucuronidase co-exposure, to recreate in situ deconjugation. For breadth, deploy RNA-seq in bulk and, where heterogeneity is consequential, single-cell RNA-seq, using time-stamped sampling: seconds to minutes for reactive cues best captured by nascent transcription assays (PRO-seq or SLAM-seq), 15, 60, and 240 min to track kinase-driven programmes, and 6–24 h to resolve microbiota-derived effects. Complement these with ATAC-seq and targeted ChIP-seq or ChIP-qPCR (e.g., NRF2, p65, AhR, TFEB, PPARγ, ERβ) to couple chromatin accessibility and factor occupancy to expression [114].

Report dose as unbound concentration, stratify by metabotype and transporter competence, control for matrix effects and cell-health drift, and integrate with CETSA/TPP plus PTM proteomics to close the engagement → PTM → gene expression loop [115].

### 5.5. Physiological Exposure Systems

Recreate physiological delivery. Use transport-competent, polarised epithelia (Caco-2/TC7, MDCK-II, primary intestinal) with verified ABCC2/ABCG2/ABCB1 and vectorial transport, for liver, sandwich-cultured primary hepatocytes forming canaliculi and maintaining MRP2/BCRP/P-gp. Dose apical vs. basolateral to mimic lumen vs. blood, and report TEER, paracellular permeability, and ABC function with probe substrates ± inhibitors/CRISPR. When possible, use gut–liver micro-physiological systems or perfused 3D organoids with defined flow/shear, bile-mimetic compartments, and EHC (enterohepatic circulation) surrogates (recirculating loops) [116].

### 5.6. PK-Informed Designs

Design experiments that mirror when/where/how much each metabolite arrives: dose at physiologically relevant unbound levels and honour biphasic timing (early host conjugates, minutes–hours, late microbiota products, 6–24 h). Time-stamp sampling (seconds-minutes, 15/60/240 min, 6–24 h). Quantify nominal vs. free, intracellular unbound, and unbound AUC, explicitly correcting for protein binding, plastic adsorption, and medium redox capacity [117].

## 6. Translation and Precision Nutrition

Translation begins with phenotyping: baseline and postprandial metabolomics of positional conjugates and microbiota-derived products (urolithins, γ-VLs, equol), paired with transporter genetic panels and stool β-glucuronidase activity, assigns each individual to an exposure architecture, early conjugate-routed or late microbiota-dependent, that predicts which TF/proteostasis modules are tractable [44,45]. It is important to distinguish what is currently clinically implementable from what remains aspirational. Approaches that are analytically accessible today include: urolithin A and equol metabotyping from spot urine using validated LC-MS/MS assays (costs < EUR 50 per sample at research scale); ABCG2 rs2231142 genotyping from buccal swabs; and stool β-glucuronidase activity measured by colourimetric or fluorogenic substrate assays. In contrast, PBPK-guided individual dose optimisation, tissue-specific transporter stratification, and companion diagnostics for ABCB1/ABCC2 polymorphism panels remain largely research-grade tools that are not yet validated for clinical regulatory decision-making and would require formal analytical validation, cost-effectiveness analysis, and regulatory guidance before routine implementation. Microbiome co-interventions, prebiotics, producer probiotics, and urolithin A as postbiotic, are commercially available but currently lack the evidence base to justify metabotype-guided prescription. These distinctions are critical for honest communication with clinicians and for designing future pragmatic trials that can be realistically conducted at scale [44,45].

Formulation should use PBPK-guided dosing at physiological nM–low-µM free concentrations, with safety built in by monitoring GSH/GSSG, NQO1 tone, and transporter liabilities. Trials should be powered for time-by-metabotype interactions, with a lean companion diagnostic (urolithin A glucuronide + 3′-γ-VL glucuronide + S-equol, stool β-glucuronidase, ABCG2 genotype) sufficient to partition responders toward NRF2/TFEB versus PPAR/AhR programmes. Microbiome co-interventions, prebiotics, producer probiotics, and urolithin A as postbiotic, offer additional levers. In sum, precision nutrition for polyphenols is exposure engineering: phenotype, formulate, time, and titrate [118,119].

## 7. Gaps, Controversies, and Research Agenda

Despite mounting support for a metabolite-centric framework, the literature still leans on aglycone pharmacology at supraphysiological doses and in non-polarised culture systems. This practice obscures the contributions of authentic conjugates and microbiota-derived products, inflates effect sizes, and sustains broad, non-specific claims of pan-assay activity [120].

The foremost gap is exposure provenance: too few studies report isomer identity, unbound concentrations, intracellular free levels, or losses to serum proteins and plastics, and almost none benchmark dosing against bile, luminal, or portal-plasma ranges. Without these anchors, neither negative nor positive findings can be compared across laboratories or translated [95].

A second point of contention concerns spatial gating versus systemic concentration. Apparent paradoxes, such as robust ARE activation despite low plasma aglycone, are largely reconciled by vectorial transport and β-glucuronidase hubs, yet direct measurements of conjugate-to-aglycone flux at relevant surfaces remain rare [19].

We still lack comprehensive atlases of β-glucuronidase activity, encompassing microbial GUS and host GUSB, across tissues, disease states, and pH-defined microenvironments, as well as standardised assays to quantify perimembrane microbursts and their oxidative conversion to o-quinones and H_2_O_2_ in vivo. Equally important, the field needs consensus on when and where quinone signalling is adaptive versus harmful, as proposed safety windows defined by NQO1 tone, glutathione capacity, and transporter status remain largely hypothetical and unmeasured in humans; until validated, these concepts should be treated as working hypotheses rather than established safety criteria [121].

Third, transporter biology remains under-integrated. Although MRP2/BCRP/P-gp routing is central to this framework, most models fail to verify ABC expression and function, neglect MRP3-mediated back-export, and overlook SLC uptake pathways that shape organelle access. ABC polymorphisms, diet-drug interactions (e.g., inhibition of ABCG2/ABCB1), and disease-driven remodelling of transporter capacity can invert tissue gradients, yet these variables are rarely stratified in experiments or trials [122].

Fourth, metabotype is often invoked but rarely measured with standardised tools. We still lack rapid assays to classify urolithin, γ-VL, and equol producer status, and we have only a partial grasp of how antibiotics, PPIs, fibre matrices, and pre/probiotics remodel these phenotypes over weeks to months. Most critically, the causal microbes and gene clusters responsible for γ-VL production remain under-resolved [123].

Methodologically, chemo-proteomic interrogation of reversible electrophiles remains difficult: competitive ABPP can overlook rapid on/off adducts, nucleophile-trapping approaches lack standardisation, and robust linkage of site occupancy to function at scale is still the exception [124].

Thermal profiling via CETSA or TPP is a powerful approach, yet it remains underused for protein interfaces and higher-order assemblies, where weak interactions can impart only subtle shifts in stability. Downstream, PTM time series are seldom synchronised to the appropriate early (seconds–minutes, 15/60/240 min) and late (6–24 h) windows, and genomic analyses too often flatten temporal structure into single endpoints [109].

To address these gaps, we propose a research agenda grounded in exposure realism and causal inference:

(1) Develop standardised reference panels of authentic metabolites, including positional glucuronides and sulphates, O-methylated forms, γ-VL isomers, the urolithin series, and equol enantiomers, accompanied by open characterisation and stability documentation, and ensure broad, price-accessible distribution [51].
(2) Define clear minimum reporting standards, covering isomer identity, nominal versus free concentrations, intracellular unbound levels, medium redox capacity, transporter competence, and β-glucuronidase activity, and commit to depositing raw LC-MS and other omics datasets in accessible repositories in keeping with FAIR principles [125].
(3) Systematically map spatial exposure using MALDI- or DESI-based MSI and micro-dialysis in transport-competent models and, where ethically feasible, in humans via targeted sampling (enterostomy or ERCP) across bile, luminal, and portal compartments, with the goal of building quantitative atlases of conjugate and aglycone distributions [126].
(4) Develop comprehensive atlases of microbial GUS and host GUSB activity across health and disease, explicitly capturing pH and mucus contexts; in parallel, design and validate selective inhibitors or boosters to test the causal role of local deconjugation [127].
(5) Integrate PBPK models capturing enterohepatic cycling, apical/canalicular compartments, MRP3-mediated back-flux, and metabotype-specific late inputs; use them to calibrate dose, pulse cadence, and window-aligned sampling in preclinical and clinical studies [128].
(6) Strengthen target-engagement methodologies for reversible electrophiles and weak binders by standardising nucleophile-trapping chemistries, extending isoTOP-ABPP to capture rapid exchange, applying interface-sensitive CETSA/TPP, and deploying site-directed mutants to close the causal chain from chemistry to function [129].
(7) Conduct metabotype-stratified trials with mechanistic co-endpoints, CETSA/TPP signatures, targeted PRM panels for phosphorylation and acetylation sampled at 15, 60, and 240 min, and TF reporter or ChIP readouts at 6–24 h, and power the design to detect time × metabotype × transporter interactions rather than average effects [130].
(8) Systematically define safety windows for quinone signalling by co-phenotyping NQO1 activity and glutathione capacity alongside a panel of sentinel biomarkers that can distinguish adaptive signalling from harmful electrophilic/oxidative stress. Proposed measurable endpoints include: (a) adaptive markers, NRF2/ARE target gene induction (NQO1, HO-1, GCLM mRNA/protein), TFEB nuclear translocation, and PPAR-RE reporter activation; (b) damage markers, glutathione depletion (GSH:GSSG ratio below 100:1 as a proposed threshold for overt oxidative stress), carbonylated or ubiquitinated protein accumulation (e.g., by slot-blot anti-dinitrophenyl or K48-linked poly-ubiquitin), and γH2AX foci as a surrogate for DNA double-strand breaks; and (c) safety sentinel assays, GSTP1 adduct quantification by targeted mass spectrometry as a traceable electrophile-exposure biomarker, and NQO1 activity ratio (dicoumarol-inhibitable vs. total) to gauge quinone-reductive buffering capacity. The guiding criterion should be that ARE/TFEB/PPAR activation is achievable at exposures that do not reduce GSH:GSSG below the proposed threshold or increase γH2AX foci beyond two-fold over vehicle, with concurrent tracking markers of DNA and protein damage alongside the desired pathway activations to confirm that signal is decoupled from stress [63].

Finally, we advocate a cultural reset: retire the reflex that “high-dose aglycone equals mechanism.” The way forward is exposure engineering, delivering authentic metabolites to the right surfaces at the right times, measuring engagement where it actually occurs, and letting physiology, transport, and the microbiome define the rules [131].

Alternative explanations and practical limitations deserve explicit acknowledgement. First, measuring free intracellular metabolite levels in intact human tissue is currently beyond routine capability. Available data rely on estimates derived from plasma unbound fractions and cell-uptake models, and the actual intracellular free concentrations at target proteins may differ by one to two orders of magnitude from nominal dosing levels, which limits the confidence with which any proposed nM–µM engagement threshold can be validated in vivo. Second, tissue-specific deconjugation by β-glucuronidase is a central tenet of this framework, yet direct quantitative measurements of aglycone microbursts at relevant surfaces (gut mucosa, tumour microenvironment) in humans are technically demanding and largely absent from the literature; existing evidence is largely indirect, based on enzyme-activity assays in biopsy material and surrogate markers. Third, real-world inter-individual variability in metabotype is well-documented at the population level, but the clinical tools to rapidly and accurately classify individuals prospectively (e.g., before enrolment in a nutritional intervention) are not yet validated or commercially available at scale. Fourth, it is important to recognise that an alternative interpretation of some findings in the literature is that polyphenol-associated health benefits may be mediated by effects on the gut microbiota composition per se, rather than by metabolite-mediated transcription-factor reprogramming in host tissues, and these two explanations are not mutually exclusive. Rigorous mechanistic attribution requires study designs that can disentangle these pathways, and such designs are still the exception rather than the rule.

## 8. Conclusions

Polyphenols do not act as generic, freely diffusing antioxidants; they operate as precursors to authentic metabolites, positional glucuronides/sulphates, O-methylated forms, and microbiota-derived xenometabolites, that reach targets through anatomically constrained routes and on biphasic timelines. This exposure geometry, vectorial transport by MRP2/BCRP/P-gp, enterohepatic cycling, and β-glucuronidase hubs, creates surface-proximal microbursts of aglycone/catechol in the early window and delivers smaller, more permeable microbial products in the late window, together gating which transcription-factor and proteostasis programmes are reachable in vivo.

Mechanistically, signalling emerges from a continuum of noncovalent biasing, conditionally gated electrophile/redox relays (catechol → o-quinone), and PTM cascades (phosphorylation → acylation → proteostasis) that edit NRF2/Keap1, NF-κB/IKK, AMPK/MAPK/PI3K-Akt, SIRT1/HDACs, PPARs, AhR, and TFEB according to where metabolites accumulate and for how long. Inter-individual variability is the rule, not the exception: metabotypes and transporter states rewire exposure and thus response, explaining the discordant literature when aglycone boluses or non-polarised models are used.

Accordingly, progress depends on methods that honour exposure: dosing authentic metabolites at nM–low µM free concentrations, transport-competent systems that recreate spatial gating, time-stamped chemoproteomics, CETSA/TPP, and phospho/acetyl-proteomics aligned to early and late windows, and genomic readouts that close the engagement → PTM → expression loop.

Translation then becomes precision nutrition by exposure engineering: phenotype metabotype and transport capacity; formulate for the desired window and tissue, and titrate to safe, effective signalling ranges. The research agenda ahead, reference metabolite panels, spatial exposure atlases, metabotype-stratified trials, and quantified safety windows, will convert this framework into actionable guidance. In sum, embracing a metabolite-centric, spatiotemporal view transforms polyphenols from diffuse “antioxidants” into programmable dietary signals for targeted reprogramming of transcription factors and proteostasis in humans.

The complete pathway from dietary polyphenol intake to proteostasis reprogramming can be summarised in five sequential steps. Step 1 (intake and phase II conjugation): dietary polyphenols are absorbed and rapidly conjugated by intestinal and hepatic UGT, SULT, and COMT into position-selective glucuronides, sulphates, and O-methyl derivatives. Step 2 (vectorial transport and tissue distribution): ABC efflux pumps (MRP2, BCRP, P-gp) route conjugates apically and canalicularly, creating surface-proximal pools at barrier epithelia and in bile, while keeping systemic levels modest. Step 3 (temporal windows of deconjugation): an early window (minutes to hours post-ingestion) is defined by β-glucuronidase-mediated aglycone microbursts at gut mucosa, inflamed tissue, and tumour sites, producing catechol-to-o-quinone electrophile and H_2_O_2_ signals; a late window (6–24 h) is defined by microbiota-derived catabolites (urolithins, γ-valerolactones, equol) arriving systemically. Step 4 (target engagement): the spatiotemporally gated signals engage NRF2/Keap1, NF-κB/IKK, AMPK/MAPK/PI3K-Akt, SIRT1/HDACs, AhR, PPARs, TFEB, and ERβ/FOXO through noncovalent biasing, covalent–redox editing, and PTM cascades, with amplitude and target selectivity set by which metabolites arrive and where. Step 5 (proteostasis reprogramming): converging phosphorylation, acylation, and autophagy signals translate transient chemical events into durable transcriptional and proteostatic programmes that outlast the initiating exposure by hours to days. This five-step model, if rendered graphically, would provide a single integrative visual for both specialist and non-specialist readers and is proposed as the basis for a future graphical abstract or summary figure.

## Figures and Tables

**Table 1:** Summary of transcription-factor and proteostasis nodes: operative metabolites, temporal windows, primary mechanisms, and recommended engagement readouts. For each node, the predominant evidence base is indicated as follows: (H) primary evidence from human studies or human-derived primary cells; (A) animal in vivo models; and (C) cell-based in vitro models only. Most nodes are currently supported mainly by (C) and (A) evidence; nodes where (H) data exist are so indicated. This column reflects the state of the literature at time of writing and should be updated as translational data accumulate.

Node	Operative Metabolites	Window	Primary Mechanism	Key Engagement Readouts
**NRF2/Keap1-ARE**	Catechol glucuronides (MRP2-routed); locally regenerated catechol/o-quinone	Early (min-h); disease-site bursts	Keap1 Cys151/273/288 adduction; quinone–H_2_O_2_ relay; NRF2 stabilisation	Keap1 CETSA/TPP; ABPP Cys competition; ARE-luciferase (s-min, 15/60/240 min, 6–24 h)
**NF-κB/IKK**	Glucuronides/sulphates (noncovalent); local catechol/o-quinone (IKKβ Cys adduction)	Early; barrier/inflamed sites	Interface biasing of IKK scaffold; IKKβ Cys quinone adduction; partial p65 suppression	IKK/NEMO CETSA/LiP-MS; thiol-ABPP; NF-κB reporter + p65 translocation (min-h)
**AMPK/MAPK/PI3K-Akt**	All conjugates (early H_2_O_2_ relay); γ-VL conjugates + urolithin A (late)	Biphasic: 15–60 min + 6–24 h	Redox/electrophile-gated phospho-waves; late organelle-driven AMPK reset via mitophagy	DIA phosphoproteomics (5/15/60/240 min); ACC-P AMPK sentinel; CETSA on kinase scaffolds
**SIRT1/HDACs**	O-methylated/conjugated metabolites (indirect NAD+ + peripheral interface); urolithin A (late NAD+ boost)	Mid (1–4 h); late (6–24 h)	Peripheral-interface biasing of HDAC conformers; indirect SIRT1 via redox-NAD+; FOXO/PGC-1α deacetylation	Targeted acetyl-PRM (parallel reaction monitoring; FOXO, p65, PGC-1α); SIRT1/HDAC CETSA-TPP; locus ChIP-seq (1–4 h, 6–24 h)
**AhR**	γ-VL conjugates; urolithin A (crosstalk); equol/enterolignans	Late (6–24 h); barrier/tumour sites	Noncovalent chaperone-complex biasing; H_2_O_2_-driven c-Jun/NF-κB crosstalk; metabotype-dependent amplitude	XRE reporter; AhR CETSA; donor-stratified fermentation effluents; 6–24 h sampling
**PPARγ**	γ-VL conjugates; equol/enterolignans; urolithin A (secondary)	Late (6–24 h); intestinal/hepatic sites	Weak peripheral groove binding; AMPK-primed coactivator exchange; metabotype-dependent	PPARγ CETSA/TPP; PPAR-RE ChIP-seq; ligand reporter; donor metabotype stratification (6–24 h)
**TFEB/autophagy-mitophagy**	All conjugates (early AMPK/mTORC1 input); urolithin A (exemplar late driver)	Biphasic: early priming + late (6–24 h) proteostasis	AMPK/mTORC1 → TFEB dephosphorylation; lysosomal biogenesis; selective mitophagy (metabotype-dependent)	TFEB reporter/nuclear translocation; tandem mRFP-GFP-LC3 flux; p62 turnover; CETSA (15/60/240 min + 6–24 h)
**ERβ/FOXO**	Equol/enterolignans (ERβ); SIRT1-upstream cues from γ-VL/urolithin A (FOXO deacetylation)	Late (6–24 h); equol-producer dependent	ERβ ligand-binding + coactivator recruitment; SIRT1-FOXO deacetylation (Lys242/245); PPAR/NRF2 crosstalk	Enantioselective equol assay; ERE-reporter + ERβ ChIP; FOXO acetyl-PRM; equol-producer stratification

## Data Availability

No new data were created or analyzed in this study. Data sharing is not applicable to this article.
